# Short chain fatty acids: the messengers from down below

**DOI:** 10.3389/fnins.2023.1197759

**Published:** 2023-07-06

**Authors:** Virginie Mansuy-Aubert, Yann Ravussin

**Affiliations:** ^1^Department of Biomedical Sciences, Faculty of Biology and Medicine, University of Lausanne, Lausanne, Switzerland; ^2^Laboratory of Energetics and Advanced Nutrition (LEAN), Department of Endocrinology, Metabolism and Cardiovascular Systems (EMC), Faculty of Science and Medicine, University of Fribourg (UNIFR), Fribourg, Switzerland

**Keywords:** short chain fatty acid, gut brain axis, metabolism, butyrate, propionate, acetate

## Abstract

Short-chain fatty acids (SCFAs), produced by the metabolism of dietary fibers in the gut, have wide-ranging effects locally and throughout the body. They modulate the enteric and central nervous systems, benefit anti-inflammatory pathways, and serve as energy sources. Recent research reveals SCFAs as crucial communicators between the gut and brain, forming the gut-brain axis. This perspective highlights key findings and discusses signaling mechanisms connecting SCFAs to the brain. By shedding light on this link, the perspective aims to inspire innovative research in this rapidly developing field.

## Background

**Figure 1:**
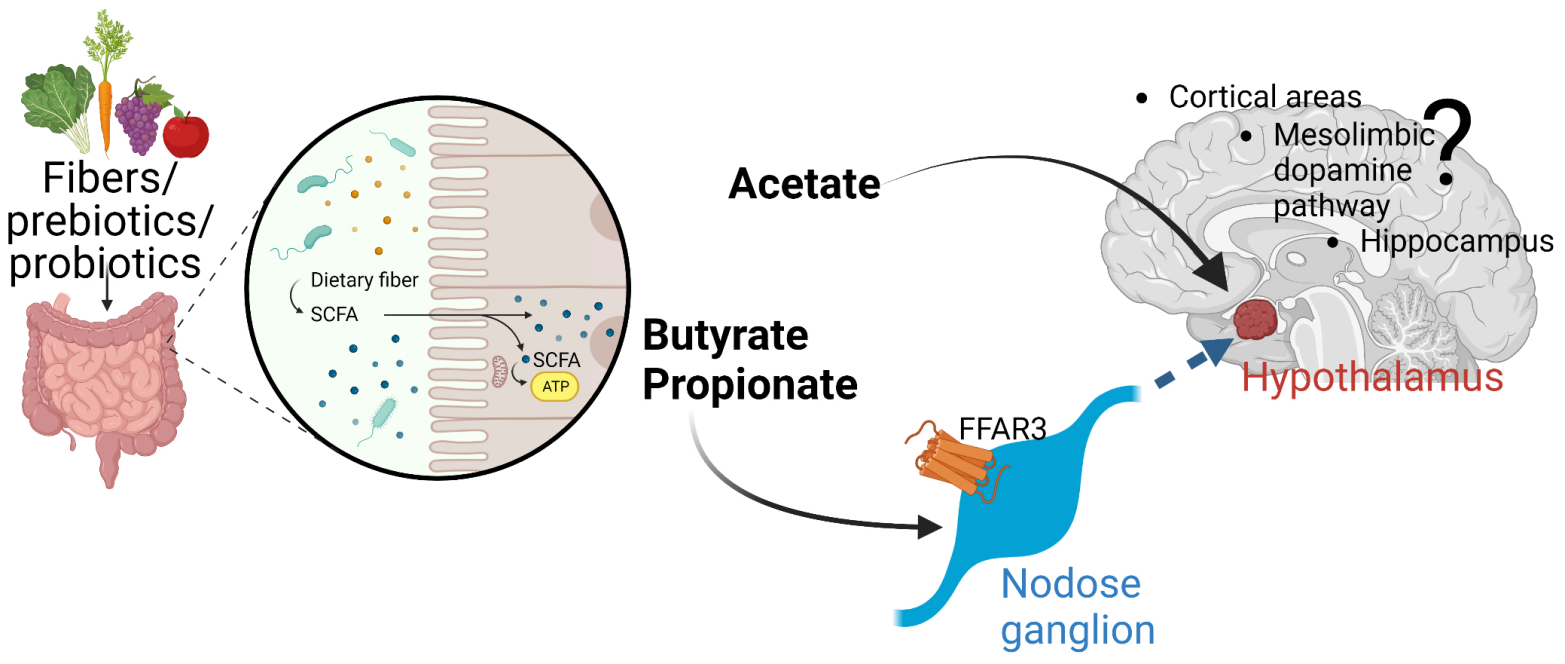
The three major SCFA (acetate, propionate, butyrate) are produced by fermentation especially in the colon of the digestive tract. While have local effects, evidence is emerging that these SCFA can either directly affect various parts of the brain (e.g., hypothalamus by acetate) or indirectly by signaling through afferent neuronal pathways (through nodose ganglia). FFAR3 seems to be an important receptor through which SCFA can signal. More work must be conducted to further elucidate these afferent pathways and their brain effects. Created with Biorender.Com.

Short-chain fatty acids (SCFAs) are a group of organic compounds produced when dietary fibers (complex carbohydrates not digested by human enzymes) are metabolized by specific bacteria found within the intestinal tract. Acetic acid, propionic acid, and butyric acid are the three most common SCFAs produced (representing ∼60, 25, and 15%, respectively, in humans; Figure 1; Cummings et al., 1987).

SCFAs have multiple effects both locally and more distally (Koh et al., 2016). They can act locally through the enteric nervous system, can modulate the central nervous system (CNS) by affecting afferent pathways to the brain, can directly affect intestinal epithelium anti-inflammatory pathways with clear benefits in numerous acute and chronic disease states, as well as serve as metabolic precursors for oxidation thereby providing energy for ATP production. Estimates suggest they are responsible for 5–15% of total caloric requirements while providing 60–70% of colonic epithelial energy in humans (Bergman, 1990; Donohoe et al., 2011). Recent scientific advances have uncovered important metabolic and cognitive consequences of SCFA that go beyond a purely energetic contribution and they are now considered a major communication link between the gut and the brain (i.e., gut-brain axis) (O'Riordan et al., 2022). Numerous recent reviews have reunited parts of these emerging roles in more detail (Astbury and Corfe, 2012; Kuwahara, 2014; Natarajan and Pluznick, 2014; Miyamoto et al., 2016; Sivaprakasam et al., 2017; de la Cuesta-Zuluaga et al., 2018; Cryan et al., 2019; Hernández et al., 2019; Sherwin et al., 2019; Jaggar et al., 2020; Long-Smith et al., 2020; Rea et al., 2020; Ramos Meyers et al., 2022; Rekha et al., 2022).

In this short perspective, we will provide a summary of the major findings to date and then discuss novel potential signaling mechanisms that link SCFAs to the brain as well as discuss what scientific approaches can be used to more fully evaluate the significant role SCFAs play in connecting the gut to the brain. We hope that this perspective will spur innovative approaches to better characterize the important link between the gut and the brain.

## SCFA – quick overview

Three major SCFA are acetate, propionate, and butyrate and each has multiple described effects. These SCFAs are important metabolites produced by gut microbiota that play a crucial role in maintaining host health. Acetate is a two-carbon SCFA that serves as an important energy source for host cells, particularly in the brain and peripheral tissues (Belanger et al., 2011). In addition to its role in energy metabolism, acetate has anti-inflammatory properties (Vinolo et al., 2011), reduces appetite and food intake in rodent models when administered by i.p. injection (Frost et al., 2014), and protects the gut epithelium by increasing the expression of tight junction proteins thereby reducing gut permeability, for example partly protecting mice against colitis (Laffin et al., 2019). Propionate, a three-carbon SCFA, regulates the release of gut hormones, such as peptide YY and glucagon-like peptide-1 (Psichas et al., 2015), and human colon delivery of propionate significantly reduces weight gain by increasing postprandial plasma PYY and GLP1 (Chambers et al., 2015). Studies in rats have found that propionate can increase activity in brain regions that are involved in the regulation of food intake and changes reward to high energy foods in humans (de Vadder et al., 2014; Byrne et al., 2016). It can reduce inflammation in the gut (Vinolo et al., 2011) and can regulate the expression of tight junction proteins in the gut (Ma et al., 2012), helping to maintain gut barrier function and preventing the entry of harmful substances into the bloodstream in part via increasing mucin production (Willemsen et al., 2003). Butyrate, a four-carbon SCFA, has potent anti-inflammatory effects, regulates gut permeability *in-vitro* (Peng et al., 2009), and can modify gene expression through epigenetic mechanisms (Govindarajan et al., 2011). Butyrate has been hypothesized as playing a potential role in the regulation of the gut-brain axis potentially via increases in production of colonic serotonin, a neurotransmitter important for regulating mood and behavior (Fukumoto et al., 2003; Yadav et al., 2013; Stilling et al., 2016). Furthermore, research conducted on rodents has proposed that butyrate might exhibit antidepressant-like properties by augmenting the expression of brain-derived neurotrophic factor (BDNF), a protein crucial for neuronal growth and survival (Intlekofer et al., 2013). Additionally, butyrate can regulate energy metabolism by increasing the production of ketone bodies, especially beta-hydroxybutyrate via increases in FGF21(Li et al., 2012), which can serve as an alternative energy source for the brain. Collectively, SCFAs are important in maintaining gut barrier function, regulating host metabolism, and modulating the gut-brain axis, with potential therapeutic applications in a range of health conditions.

## SCFA and systemic metabolism

SCFAs influence multiple metabolic parameters including energy expenditure and alterations in glucose and lipid metabolism. Butyrate increases insulin sensitivity by stimulating the production of insulin-sensitizing hormones like GLP1 and reducing inflammation in adipose tissue (Gao et al., 2009; Yadav et al., 2013). SCFAs also influence lipid metabolism by modulating the expression of genes involved in lipid metabolism (e.g., PPARᵧ) in the liver and adipose tissue (Morrison and Preston, 2016). Acetate and propionate can be used as substrates for gluconeogenesis (the production of glucose from non-carbohydrate sources), while butyrate can be used as an energy source by colonic epithelial cells (Donohoe et al., 2011). Furthermore, SCFAs influence appetite regulation and energy balance in rodents and humans (as detailed below) likely preceding changes in systemic metabolism. Propionate, in particular, can stimulate the release of appetite-regulating hormones, such as peptide YY (PYY) and glucagon-like peptide-1 (GLP-1), which can reduce food intake, increase satiety and modify glucose homeostasis (Tolhurst et al., 2012; Psichas et al., 2015; Caengprasath et al., 2020). Overall, while the mechanisms by which SCFAs affect metabolism are still under investigation, there is growing evidence that they have beneficial effects on glucose and lipid metabolism, as well as appetite regulation and energy balance as a whole.

## SCFA and neuropathy

Recent publications indicate that gut microbiota may be linked to peripheral neuropathy in obesity (Bonomo et al., 2020; Guo et al., 2023). Data published suggest that microbiome transplantation from lean to obese mice may alleviate pain and increases nerve survival and/or growth via short chain fatty acids (e.g., butyrate) (Bonomo et al., 2020). Recently, *in-vitro* studies demonstrated a potential antioxidative and neurodegenerative effect of propionate in the peripheral nerve system suggesting that propionate could be used in immune-mediated neuropathies (Grüter et al., 2023). Some SCFA have been demonstrated to cross the blood brain barrier in rats (Oldendorf, 1973) and in humans (Bachmann et al., 1979), possibly due to high expression of their transporter, monocarboxylate transporters (MCTs) in blood cells and astrocytes (Bachmann et al., 1979; Dalile et al., 2019). MCTs are also expressed in the PNS, especially MCT1 in dorsal root ganglions (DRG), Schwann cells (SC) and perineurial cells (Feldman et al., 2017). In a model of crush injury, MCT1-deficient mice showed delayed nerve regeneration, providing evidence that SCFA, which can be used by and shuttled between cells of the PNS, are essential for axonal regrowth (Morrison et al., 2015).

## SCFA and neural control of energy balance

SCFAs have been found in appreciable amounts in the cerebrospinal fluid suggesting they can cross the blood–brain barrier and concentrations of SCFAs have been linked to alterations in mood, behavior, and cognitive function [as reviewed in O'Riordan et al. (2022)]. As described above, the gut microbiota and its SCFA have also a role in regulating host metabolism. The hypothalamus is critical for adapting energy and glucose homeostasis by integrating sensory, hormonal and/or gastrointestinal nutrient status (Gupta et al., 2020). Interestingly, taxonomy diversity of the gut microbiota correlates with changes in hypothalamic brain structure in obese and lean individuals suggesting that microbiota can regulate energy balance via an action on hypothalamus (Fernandez-Real et al., 2015). Various studies have used SCFA administration (both centrally and peripherally) and shown that SCFA can reduce energy intake via direct effects on hypothalamic neurons (acetate) or indirectly through fibers that innervate the hypothalamus (propionate and butyrate; de Vadder et al., 2014; Frost et al., 2014; Li et al., 2018; Blaak et al., 2020; Cook et al., 2021; Cook and Mansuy-Aubert, 2022). Oral butyrate delivery to mice reduces food intake and decreases c-fos in hypothalamus (Li et al., 2018). Dietary propionate alters food intake in mice but not in rats and in all rodents induces c-fos in hypothalamus and parabrachial nucleus (de Vadder et al., 2014; Cook et al., 2021). Both ICV and IP injection of acetate in mice decreases food intake and changes hypothalamic activity and gene expression (Frost et al., 2014) suggesting both direct CNS and indirect effects. In some of these studies, SCFA treatment also modified glucose sensing and glucose homeostasis (de Vadder et al., 2014; Koh et al., 2016). The gut microbiota has been shown to also modulate neuronal and microglia activity in the brainstem. Indeed, c-fos in the NTS can be induced by an injection of LPS and butyrate treatment decreases this activation with a concomitant decrease in food intake (Chaskiel et al., 2016; Li et al., 2018).

The use of conventional and germ-free models identified that the gut microbiota impacts the function of others brain areas such as the hippocampus [regulating memory, learned experience and energy status (Luczynski et al., 2016; Liu and Kanoski, 2018; Luo et al., 2018; Tang et al., 2020; Liu et al., 2021; Yu et al., 2021; Rei et al., 2022; Salami and Soheili, 2022; Li et al., 2023)] and cortical areas [regulating motivation, reward, and impulsive behavior (Gacias et al., 2016; Hoban et al., 2016; Chen et al., 2019; Bai et al., 2021; Wang et al., 2022a,b)]. To our knowledge, there are no convincing studies implicating SCFA with the function of these brain areas yet this remains to be further explored.

## SCFA and the vagus

The vagus nerve is a cranial nerve that originates in the brainstem and innervates many organs in the body, including the gastrointestinal tract, and there is a body of evidence that it mediates a gut microbiota/brain interaction, however the precise mechanism is not defined yet. In recent years, a lot of effort has been directed towards characterizing the anatomical and molecular signatures of vagal afferents. Studies have shown that cholecystokinin (CCK) Receptor or GLP1R-expressing vagal afferents relay anorexigenic signals to the brain that lead to meal termination. Interestingly, a study found that inhibition of GLP1R vagal afferents elevates blood glucose levels independently of food intake (Borgmann et al., 2021). The afferents that express GLP1R extend projections into the muscular layer of the digestive tract and respond to stretch.

Propionate can induce GLP1 secretion by enteroendocrine cells via the receptor FFAR2 but by unidentified mechanisms (Tolhurst et al., 2012; Psichas et al., 2015; Caengprasath et al., 2020). Treatment of enteroendocrine cells with either butyrate or propionate and changes in gut microbiota modifies the expression of umami or sweet taste receptors suggesting that SCFA may potentiate sensitivity to nutrients (Swartz et al., 2012; Bernard et al., 2019; Shackley et al., 2020). Intraperitoneal injection of SCFAs reduced food intake especially in fasted mice, blunted by capsaicin-induced sensory denervation (de Vadder et al., 2014; Goswami et al., 2018). In addition to its effects on gut hormone release, SCFAs directly activate the vagus nerve. For example, butyrate has been shown to increase the firing rate of vagal afferent neurons, which can transmit signals from the gut to the brain (Lal et al., 2001). Studies have shown that SCFAs can stimulate the release of the hormone cholecystokinin (CCK), which can activate the vagus nerve and increase the feeling of fullness after a meal.

FFAR3 is expressed on vagal afferents coming from the gut (Cook et al., 2021). Vagal-FFAR3KO resulted in larger meal sizes, as well as increased food intake during fasting/refeeding challenges. Furthermore, the suppressive effect of propionate supplementation on appetite was absent in vagal-FFAR3KO mice. Sequencing approaches suggested that the interaction between FFAR3 signaling, and cholecystokinin (CCK) and leptin receptor pathways in the nodose ganglia leads to modifications in food intake (Cook et al., 2021). In the past, *in vitro* and whole body KO studies implicated FFAR3 in inflammation (Kim et al., 2013; Trompette et al., 2014), metabolic disorders (Samuel et al., 2008; Bellahcene et al., 2013; Tang et al., 2015), and in promoting sympathetic tone (Inoue et al., 2012; Won et al., 2013).

The relationship between SCFAs and the vagus nerve is intricate and multifaceted, necessitating further research to comprehensively understand their interactions. However, it is evident that SCFAs have the ability to influence vagal function, highlighting a crucial aspect of gut-brain communication. To date, the only established direct connection between SCFAs and the vagus nerve is through the SCFA receptor FFAR3, although confirmation of the exact link between SCFAs and FFAR3 is still needed. Nevertheless, it is highly likely that SCFAs also exert their impact on gut-brain communication through indirect pathways.

## SCFA and local gut effects

SCFAs interact with histone deacetylases (HDACs; Fellows et al., 2018), enzymes that play a role in the epigenetic regulation of gene expression. SCFAs can inhibit the activity of HDACs, leading to changes in gene expression locally within the epithelial cells of the gut. This interaction between SCFAs and HDACs is important because it has been associated with various biological effects. For example, butyrate has been shown to regulate the expression of genes involved in cell proliferation, differentiation, and apoptosis in cancer cells, leading to growth inhibition and cell death, likely playing a role in the protection against certain cancers when consuming a diet rich in dietary fibers (Mariadason, 2008). Butyrate has also been shown to reduce inflammation by inhibiting the production of pro-inflammatory cytokines and chemokines and it has been shown to suppress motility of colorectal cancer cells providing a potential clue to its anti-cancer effects (Li et al., 2017). Overall, the interaction between SCFAs and HDACs is an important mechanism by which SCFAs can regulate gene expression and have various biological effects, including anti-inflammatory and anti-cancer effects. Further disentanglement of the local vs. systemic effects of SCFA will provide ample avenues of future research.

## Influencing microbiota to affect health

Diet is a readily modulable factor that can have important effects on numerous physiological and psychiatric outcomes. Here we briefly outline some prebiotics and probiotics that may be used to specifically affect the microbiome and its released metabolites thereby impacting health.

### Prebiotics

Although there are some products that directly provide short-chain fatty acids (SCFAs) in the form of salts, dietary supplements that promote the growth of SCFA-producing bacteria in the gut resulting in the local production of SCFA are far more prevalent. These supplements, commonly known as prebiotics, contain digestion-resistant fibers that can only be fermented by gut bacteria in the colon, resulting in the production of SCFAs. Common prebiotic supplements include fructooligosaccharides (FOS), galactooligosaccharides (GOS), inulin, and resistant starches. These prebiotics are naturally found in many foods, including chicory root, Jerusalem artichokes, onions, garlic, bananas, oats, and barley and companies have now started producing mixes of such supplements with the goal to increase the number and concentration of SCFA-producing bacteria in the gut. A few examples of how such prebiotics can affect microbiota are given below.

Administration of oligofructose or inulin, restores populations of *Akkermansia muciniphila* in diet induced obese (DIO) mice and normalizes body weight (BW) (Everard et al., 2013). The mechanisms by which prebiotics improve glucose homeostasis and energy balance is not defined but there is a body of evidence that implies an increase in SCFAs. SCFAs are monocarboxylic acids produced by fermentation of fibers by various genera of bacteria including *Lactobacillus*, *Bifidobacterium*, *Prevotella*, and *Bacteroides* (Markowiak-Kopeć and Śliżewska, 2020) and these species are under-represented in western-diet-fed mice but increased after fecal transplantation from lean to obese mice, inducing an improvement of glucose homeostasis (Koh et al., 2016; Dalile et al., 2019; Bonomo et al., 2020; Jameson et al., 2020; Cook et al., 2021). In a study by DeVadder and colleagues, fiber-derived propionate improved glucose tolerance and insulin sensitivity (de Vadder et al., 2014). Interestingly, these benefits of propionate were lost when the rats underwent a procedure to destroy sensory neurons innervating the portal vein.

### Probiotics

Although there is growing debate as to whether fetuses are within a sterile womb or not (Stinson et al., 2019), the fact that the human microbiota changes over time in relationship to a host of different environmental factors (e.g., use of antibiotic, diet, age etc.) shows that we can directly affect the bacteria as well as reseed them in our guts once our normal microbiota has been depleted (e.g., following antibiotics). Foods containing active live bacteria have been consumed for centuries (e.g., yoghurts) and there are now various manners in which to use live bacteria to influence the prevalence of certain strains. Probiotics are live microorganisms that, when consumed in adequate amounts, provide health benefits to the host. Certain strains of probiotics have been shown to increase the abundance of SCFA-producing bacteria in the gut, leading to higher levels of SCFAs. For example, the probiotic strain *Bifidobacterium lactis* has been shown to increase the production of butyrate (Riviere et al., 2016). Other strains, such as *Lactobacillus acidophilus* and *Lactobacillus plantarum*, have been shown to increase the production of propionate and acetate, respectively (Wullt et al., 2007; Markowiak-Kopeć and Śliżewska, 2020). In addition to preventing weight gain, probiotics (live bacteria) have been found to promote weight loss in mice fed a high fat diet for 12 weeks (Osterberg et al., 2015). Based on many independent data, microbiome alterations appear sufficient to alter caloric intake. It’s important to note that the effectiveness of probiotics in increasing SCFA production may depend on various factors, including the specific strains used, the dose, and the individual’s gut microbiome. Additionally, the benefits of probiotics may not be sustained over the long term, as the introduced strains may not colonize the gut permanently. It is important to comprehend the implications of SCFA in health and metabolism. However, to date, there is no direct evidence linking SCFA to the beneficial effects of pro/prebiotics. Nevertheless, continued efforts looking at how introduction of live bacteria can affect overall gut microbiota composition/health and positively impact human well-being will likely produce positive breakthroughs.

## Conclusion

In the past decade, a massive amount of studies - using extensively germ-free or fecal transplantation models - were useful in drawing primary correlative conclusions between the gut microbiota and several neurological conditions (Sittipo et al., 2022). In the last few years, a greater amount of attention has been placed on finding mechanisms underlying the microbiota-gut-brain connection at a molecular level. Much evidence shows that microbes signal to the brain in part via short-chain fatty acids. The therapeutic translation of SCFA needs deep multi-organism studies evaluating such mechanisms so that they can be better understood and potentially harnessed to positively affect human health. For example, it is important to evaluate how SCFA regulates CNS cell activity, behavior and physiology using genetically modified models and site-specific injections that target these receptors. Although considerable efforts over the past two decades have evaluated the microbiota-gut-brain axis in rodents using correlative studies, it is crucial we develop mechanistic studies in rodents but also in other species to evaluate new drugs targets that will be the focus of large and rigorous human studies. The time and tools now available seem perfectly conducive to finally being to decipher the important link between the gut and brain and how SCFAs play a crucial role in that connection.

## Data availability statement

The original contributions presented in the study are included in the article/supplementary material, further inquiries can be directed to the corresponding author.

## Author contributions

VM-A and YR came up with the perspective, conducted the research, wrote and edited all written parts, and approved the final submission.

## Conflict of interest

The authors declare that the research was conducted in the absence of any commercial or financial relationships that could be construed as a potential conflict of interest.

## Publisher’s note

All claims expressed in this article are solely those of the authors and do not necessarily represent those of their affiliated organizations, or those of the publisher, the editors and the reviewers. Any product that may be evaluated in this article, or claim that may be made by its manufacturer, is not guaranteed or endorsed by the publisher.
